# Structural Design and Controllability of Magnetorheological Grease Buffers under Impact Loading

**DOI:** 10.3390/ma16134724

**Published:** 2023-06-29

**Authors:** Gaoyang Kong, Qing Ouyang, Hongsheng Hu, Wenfeng Xiang, Wei Zhao

**Affiliations:** 1 College of Mechanical Engineering, Zhejiang University of Technology, Hangzhou 310014, China; konggy0411@163.com; 2 College of Information Science and Engineering, Jiaxing University, Jiaxing 314001, China; zhaooweii@sina.com; 3 School of Mechanical Engineering, Nanjing University of Science and Technology, Nanjing 210044, China; 4 Taizhou Jiuju Technology Co., Ltd., Taizhou 318000, China; xiangwenfeng2005@163.com

**Keywords:** magnetorheological buffers, magnetorheological grease, buffer control, impact load buffering

## Abstract

Shock loads can pose a great threat to personnel or instruments, and efficient control of the buffering process is an effective means of reducing damage from shock energy. In this paper, magneto-rheological grease was used as the internal controllable material of the buffer to address the turbulence and settling problems of conventional magneto-rheological fluid. A bending and folding back magnetic circuit is proposed, and the magnetic circuit simulation was verified. The corresponding dynamic mechanical model was established, and the mechanical response characteristics of the buffer under impact load were also simulated dynamically. The mechanical properties of the designed and processed device were tested, and a variable current control method was used to improve the performance of the shock resistance of the buffer. The response of the magnetorheological grease buffer under different drop hammer impacts was investigated. The buffering effect and controllability of the buffer were analyzed by comparing the acceleration, velocity, and top-end cap displacement at the same drop hammer height for different current magnitudes. The results show that the buffer performance of the buffer gradually improved as the current increased. The response time of the designed new magnetorheological buffer was determined by the jump time of the peak damping force to be 9 ms. Lastly, the controllability was verified by manually and automatically adjusting the current magnitude, and the results were compared with those at 300 mm drop hammer height and 0.5 A current magnitude, and the continuous variable current control was found to be effective. This provides a feasible reference for scholars to study optimal buffer control.

## 1. Introduction

The impact of shock loads on cushioning systems has long threatened the safety of personnel or critical devices on carriers. When operating in harsh environments, large shock loads may be transmitted to the interior of the carrier, and the high-intensity loads may cause damage to the equivalent load [1,2,3]. Magnetorheological energy absorbers (MREAs) are widely used in carriers such as aircraft landing gear [4,5,6], helicopters [7,8], watercraft [9], and ground vehicles [10] that are subjected to linear reciprocating shock loads for long periods of time because of their outstanding advantages of continuously controllable rheological characteristics and fast response time of magnetic fields [11] and the magnetorheological fluid itself [12]. Since the impact conditions are often accompanied by high collision energy, the controllable damping force of magnetorheological buffers is highly demanded. The most effective way to enhance the controllable damping force range is to increase the number of turns or the number of excitation coils. Xu et al. [13] theoretically analyzed the magnetic circuit of a multistage coil magnetorheological damper and gave the calculation of the magnetic induction intensity at the effective gap of the multistage coil MR damper according to the Ohm’s law of magnetic circuit. Carlson et al. proposed a magnetorheological damper with three excitation coils [14,15]. Three sets of excitation coils were designed to be arranged on the piston, and the piston section and cylinder barrel formed multiple closed magnetic circuits; multiple axial circular damping gaps were also formed between the piston section and cylinder barrel, and this three-coil design increased its effective damping length. Although the damping force output of this magnetorheological damper is huge, its size also occupies a large space; hence, it is only widely used in the seismic field. Bai et al. proposed an inner bypass annular valve-type magnetorheological damper composed of concentric tubes [16]. For conditions where the nature of the shock load was defined, the MREA could be used to provide a cushioning damping force so that the carrier could stop smoothly at the end of the stroke after fully utilizing the available stroke [17]. In order to ensure that the impact load is adequately absorbed during the buffering phase, a reasonable control of the MREA damping force is necessary [18,19,20,21,22].

Pan et al. [23] combined parametric and nonparametric models to model the nonlinear expression of displacement and velocity on the damping force using an adaptive neuro-fuzzy system, and they described the output model of the damping force with voltage and velocity using parametric methods. The new model established was used to achieve better control of the magnetorheological buffer. In a study of the shock response of magnetorheological buffers, Yancheng Li [24] established a mechanical model under shock load and performed the identification of model parameters, proposed a magnetorheological shock buffer control strategy, and designed a magnetorheological buffer shock experimental bench. However, the validation experiments were in semi-physical real-time simulation mode, and the effectiveness of the proposed model and control strategy was not really verified.

**Figure 1:**
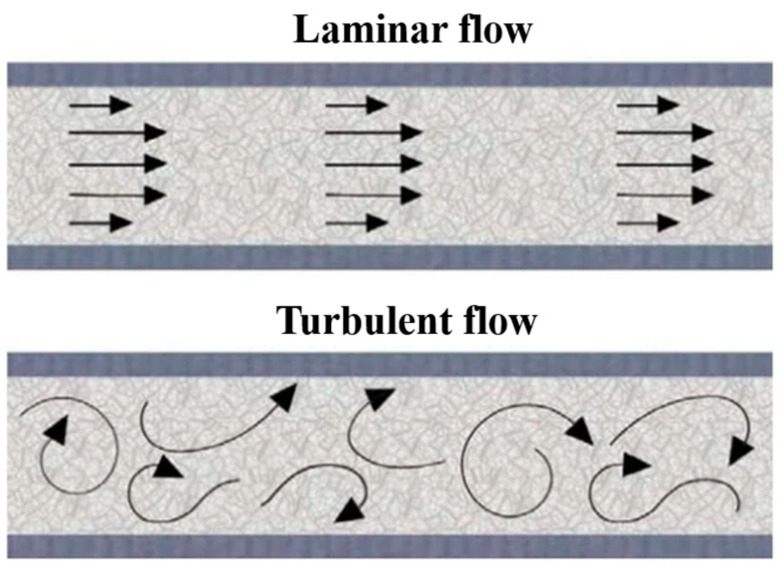
Schematic diagram of laminar and turbulent flow phenomena.

At this stage, magnetorheological (MR) materials are available in various forms such as magnetorheological fluid (MRF), magnetorheological elastomer (MRE), and magnetorheological grease (MRG). Due to the large transient impact force during buffering, the impact resistance of MR materials is required to be high. Conventional MRF materials may experience internal material volume compression, nonregular flow under severe shock loading, etc., resulting in the inability to provide a controlled damping force with a continuous, stable, and high dynamic range during impacting operation [25]. In addition, the shock buffering occurs at a much lower time than conventional vibration control, with shock response times on the order of milliseconds, although MRFs have excellent MR response capabilities, and they can respond and provide damping forces instantly at the moment of shock arrival. However, the shock load condition is often accompanied by high velocity and high shock energy, both of which will cause huge impact on the internal MR material. This can destroy the uniform arrangement of magnetic particles inside the MR material formed through the magnetization effect, as well as cause turbulence inside the MR material (as shown in Figure 1), thus affecting the damping force provided by the MR buffer and interfering with the accurate calculation of electromagnetic effect, which is not conducive to shock-resistant MR buffer control.

At the same time, the settling phenomenon of MRF is also one of the problems that need to be solved urgently. Although scholars have tried to improve or slow down the settling, coalescence, and lubrication of magnetic particles by replacing different additives, MRF will still cause different degrees of settling after a period of time. The buffer damping force provided by the MR buffer at work mainly relies on the chain of particles suspended in the base liquid due to the magnetization effect, which is continuously destroyed and reassembled, and the laminar flow phenomenon occurs inside the MRF when the piston moves. The MRF after settling will exhibit magnetic particles and base liquid delamination; at this time, the magnetic particles piled up at the bottom cannot be magnetically controlled and, thus, cannot generate the chain of magnetic particles magnetized in the damping channel when the MR device buffers. In other words, it cannot generate the buffer damping force. In addition, the settling that does not show complete solid–liquid separation (i.e., light settling) also leads to uneven distribution of magnetic particles inside the MRF, which affects the accurate control of the buffer damping force during buffer control. The MRG material can easily solve the problems of settling and turbulent flow, and is more suitable for application in impact conditions [26,27,28,29].

Therefore, in this paper, on the basis of the traditional magnetorheological buffer, a bending foldback magnetic circuit is designed using MRG as the controllable material, which effectively increases the number of available magnetic inductors in the damping channel. The internal dynamics of the MR buffer during impact are modeled. The mechanical properties of the designed and processed device are tested, and a variable current control method is used to improve the buffer performance of the buffer. The response of the new MR buffer when subjected to different drop hammer impacts is studied. The buffering effect and controllability of the MR buffer are analyzed. A feasibility analysis reference is provided for the optimal buffer control.

## 2. Magnetic Circuit Design

In order to improve the damping performance of the MR buffer in a limited space, we designed a MR buffer with a compact structure, reasonable design of each part, and full use of the magnetic circuit. Due to the electromagnetic effect, the magnetic field generated by the coil inside the magnetorheological buffer after being energized will surround the coil in a ring shape, and, when the MR buffer performs the buffering work, only the magnetic induction lines passing perpendicular to the damping channel in the ring gap will be effective.

### 2.1. Magneto-Rheological Grease Material

The MRG material used was configured by Nanjing University of Science and Technology, model MRG-76, containing 76% hydroxy iron powder rate; the main parameters are shown in Table 1. Among them, the magnetic flux saturation density performance is relatively good, which can be applied to the buffering effect under the impact working condition.

**Figure 2:**
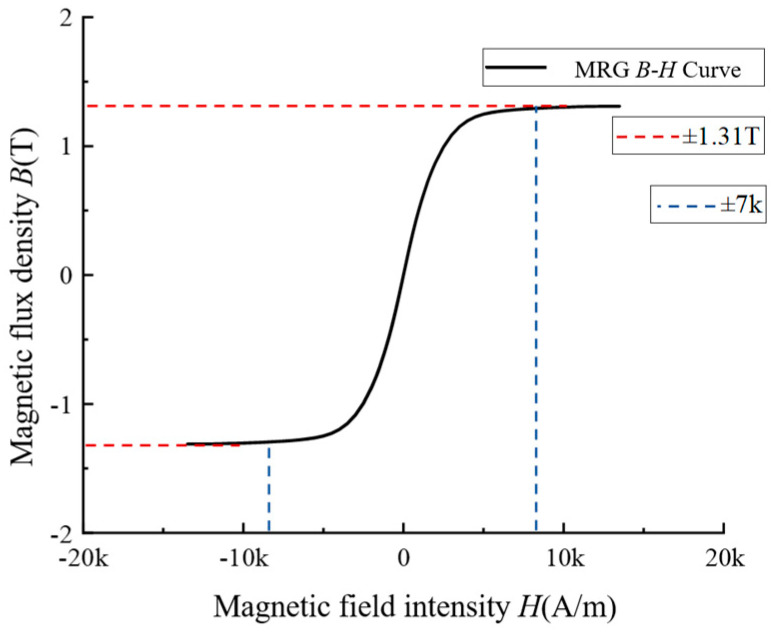
MRG B–H curve.

The relationship between the variation of magnetic flux density with magnetic field strength for MRG materials was studied. The relationship between the magnetic flux density B and the magnetic field strength H is shown in Figure 2. It can be seen that, when the magnetic field strength is ±7 kA/m, the magnetic saturation phenomenon occurs in MRG, and the magnetic flux density at this time is 1.31 T, which can meet the magnetic field strength requirement of the buffer according to the empirical value estimation. The magnetic flux density variation curve of MRG with magnetic field strength is relatively smooth, and the symmetry at both ends is also good, which can indicate that the excitation effect inside the material is more ideal and the performance is more excellent. After reaching the magnetic saturation strength, the flux density curves tend to be parallel, indicating that the magnetic saturation characteristics of the MRG material are ideal and very suitable for MR buffers under shock loads.

**Figure 3:**
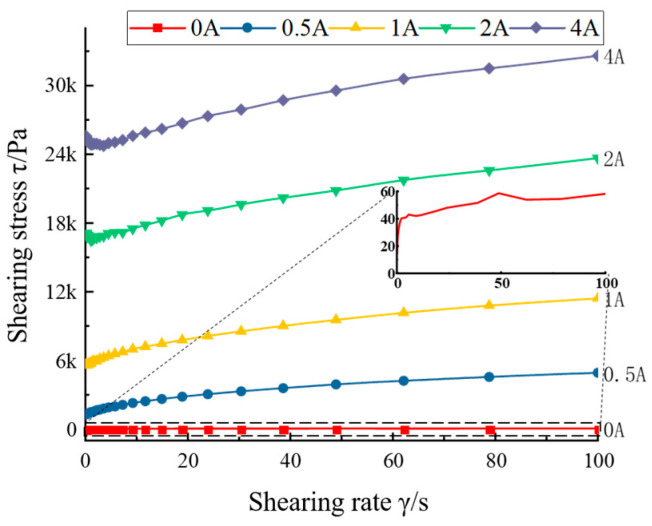
Flow curve of MRG.

**Figure 4:**
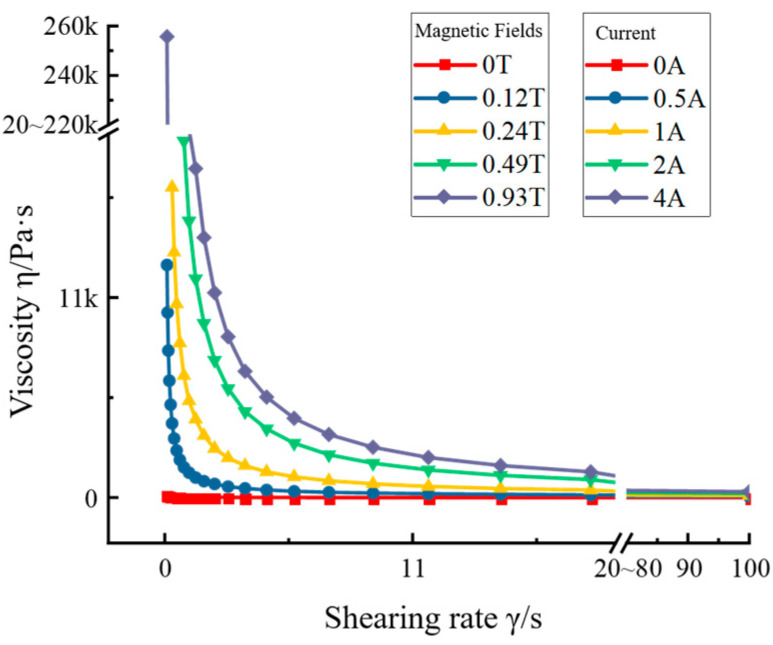
Magnetorheological grease viscosity vs. shear rate graph.

The variation of its viscosity, shear stress, and yield stress under the action of magnetic field was studied. The relationship between shear stress and shear rate is shown in Figure 3, which also reflects the flow ability of MRG. It can be seen that, when the current increased, the shear stress increased as the shear rate increased, which indicates that the MRG has good damping properties. When the current value was 0 A, the shear stress size was 40–60 Pa, which indicates that the selected MRG has good flow ability. Figure 4 shows the relationship between the flow viscosity and shear rate of the MRG material, which reflects the relationship between the flow damping force inside the fluid and the flow velocity. It can be seen that, when the current increased, the flow viscosity of MRG increased with the increase in shear rate, which indicates a good impact regulation ability.

### 2.2. Structural Form

**Figure 5:**
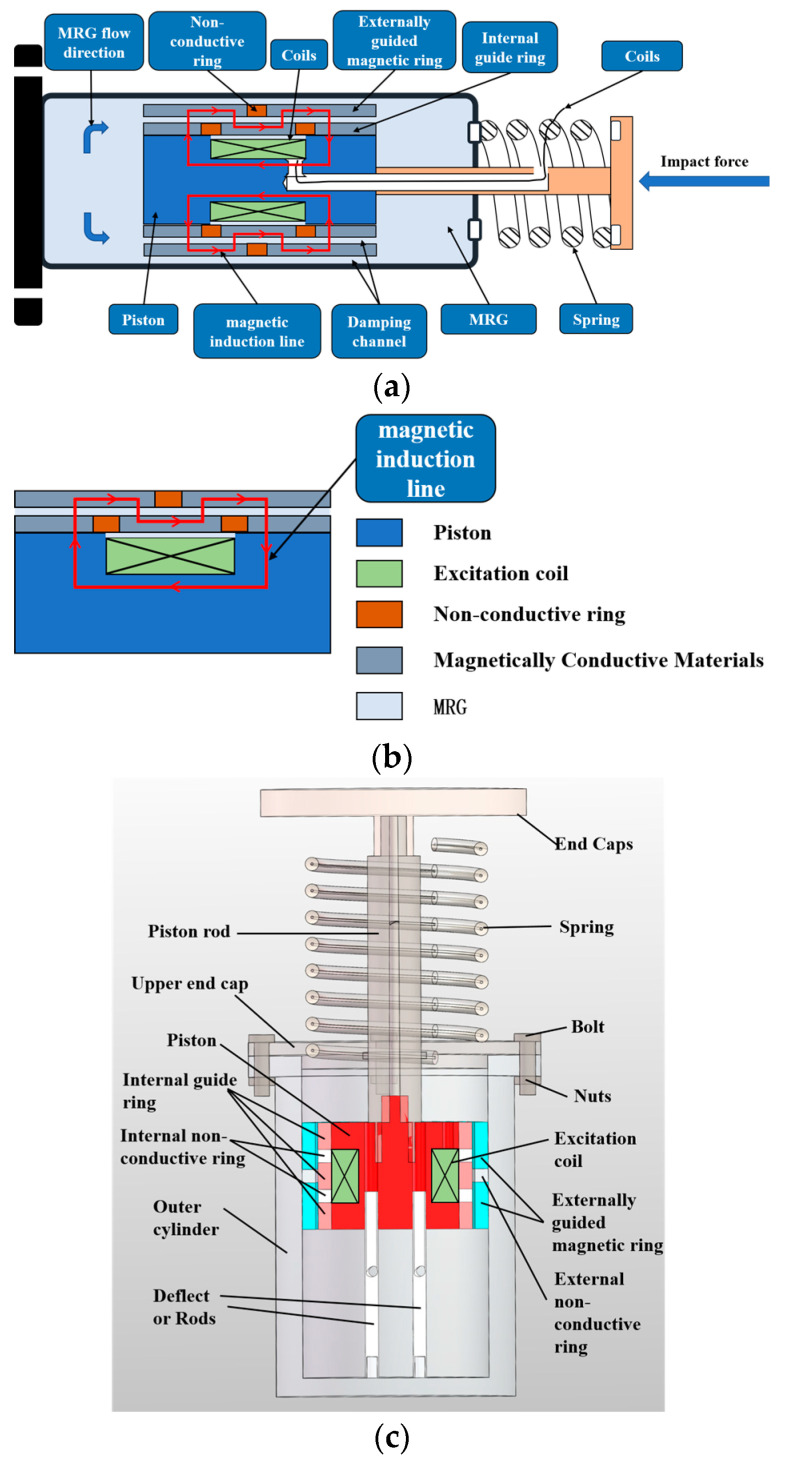
Schematic diagram of the new magnetorheological buffer structure: (**a**) schematic diagram of the structure; (**b**) schematic diagram of the magnetic circuit; (**c**) 3D structure.

An MR buffer with bending magnetic circuit operation is proposed, and the designed structure schematic is shown in Figure 5a, which realizes the bending fold back interpolation of the excitation magnetic induction lines in the piston excitation unit by two external permeable rings with three internal permeable rings, and one external nonpermeable ring with two internal nonpermeable rings. Figure 5b shows the schematic diagram of the partial structure, which can show more clearly that the magnetic induction lines are bent and folded back for interpolation with the cooperation of the permeable and nonpermeable materials. Figure 5c shows the schematic diagram of the 3D overall structure of the bending foldback magnetic circuit MR buffer. It is noteworthy that two guide rods were designed at the bottom of the buffer and inserted vertically into the main part of the piston, which can reduce the non-Newtonian effect of MRG due to impact and increase the liquid flow because of the volume compensation of the piston rod. On the other hand, it can also ensure the stability of the piston when it moves.

The specific dimensional parameters are given in Table 2.

### 2.3. Analysis of Magnetic Field Simulation Results

In the structural design, the design goal was to increase the number and frequency of magnetic induction lines passing through the damping gap as much as possible. According to this principle, a MR buffer was designed that can effectively improve the utilization of the magnetic field. The proposed magnetic circuit could be effectively shuttled through the damping gap several times with the same current through the coil, and the utilization rate of the excitation induction lines was significantly increased.

**Figure 6:**
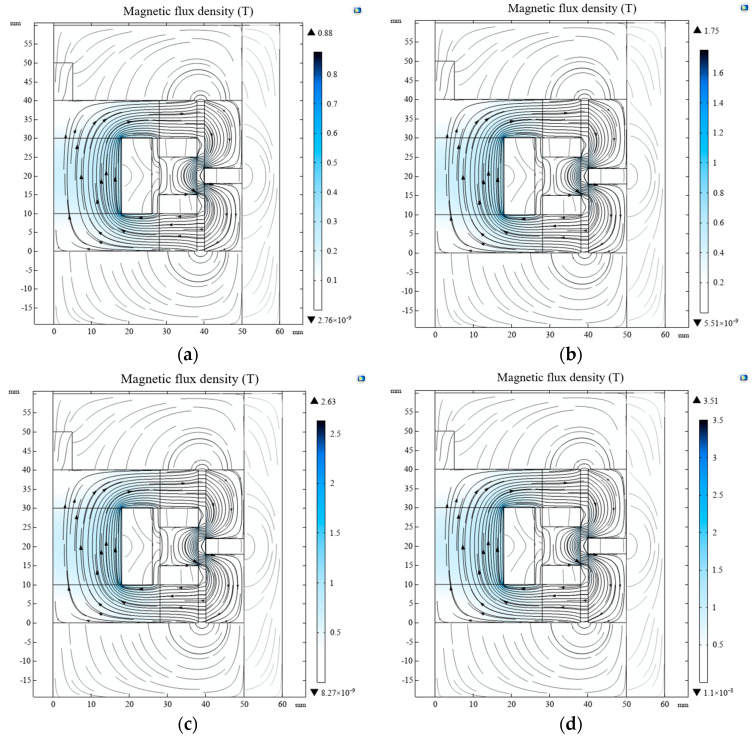
Magnetic field strength and distribution of magnetic lines of force: (**a**) 0.5 A; (**b**) 1 A; (**c**) 1.5 A; (**d**) 2 A.

Figure 6 shows the magnetic field simulation results of the new MR buffer. The liquid in the damping channel was MRG. Compared with the conventional MRF, the overall magnetic field strengths were both significantly increased, and the amount of magnetic leakage was reduced, because there were sufficient hydroxyl magnetic particles inside the MRG with excellent magnetic conductivity. When the incoming current was 2 A, the magnetic field strength at some of the corners reached 3.51 T. This value is relatively large and may affect the normal operation of the components inside the MR buffer. However, because this peak occurred at the corners of the piston and was a simulation of the magnitude of the magnetic field strength, the properties of each material were ideal, and it is difficult to see such a large value in practice. Therefore, this peak could be ignored, and the simulation could continue to the next step.

**Figure 7:**
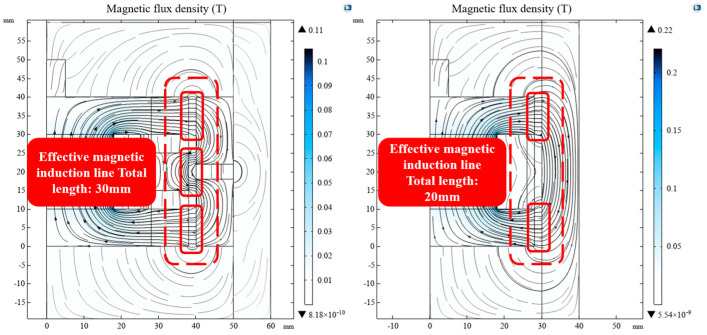
Comparison of foldback magnetic circuit and conventional magnetic circuit (0.5 A current applied).

As can be seen in Figure 7, the magnetic inductance lines interspersed in the damping channel could cause the magnetization effect of the MRG to achieve the output of the buffering damping force; thus, the number of magnetic inductance lines in the damping channel is the key to the resistance when cushioning. As can be seen from the figure, the number of magnetic inductors interspersed in the damping channel generated by the excitation of the designed bending foldback magnetic circuit was 1.5 times higher than that of the conventional magnetic circuit. This proves that the bending magnetic circuit structure with the interplay of permeable and nonpermeable rings is effective and reliable, and the designed bending foldback magnetic circuit buffer can provide a larger buffer damping force than the conventional buffer at a constant current.

**Figure 8:**
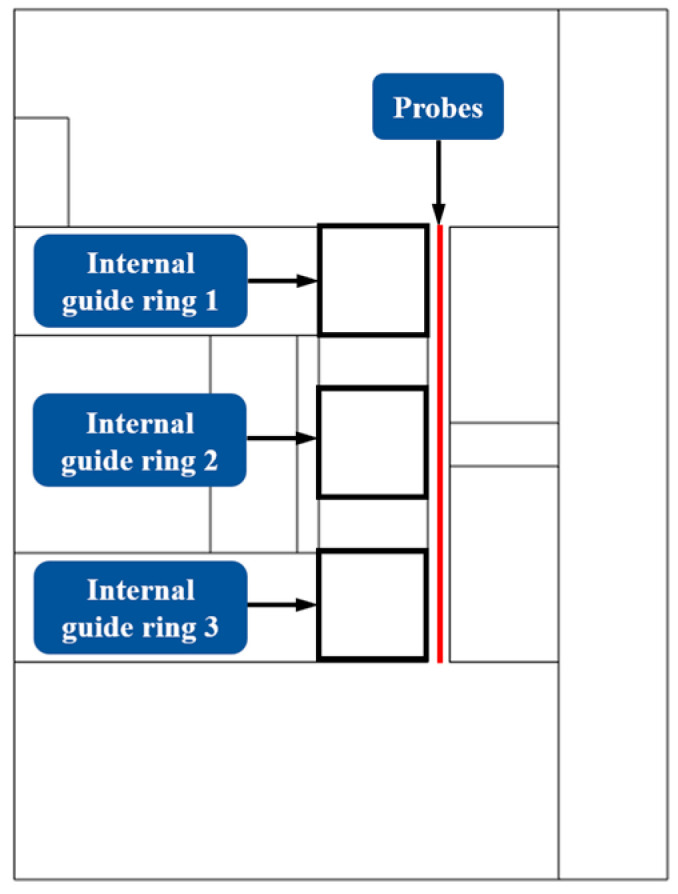
Probe position.

To further investigate the distribution of the magnetic field strength in the damping channel and to facilitate comparison with the actual measurement results, the change in its magnetic field strength under the action of the applied current was measured by adding a probe inside the damping channel. Figure 8 shows the insertion position of the measurement probe in the simulation software (comsol 5.6).

**Figure 9:**
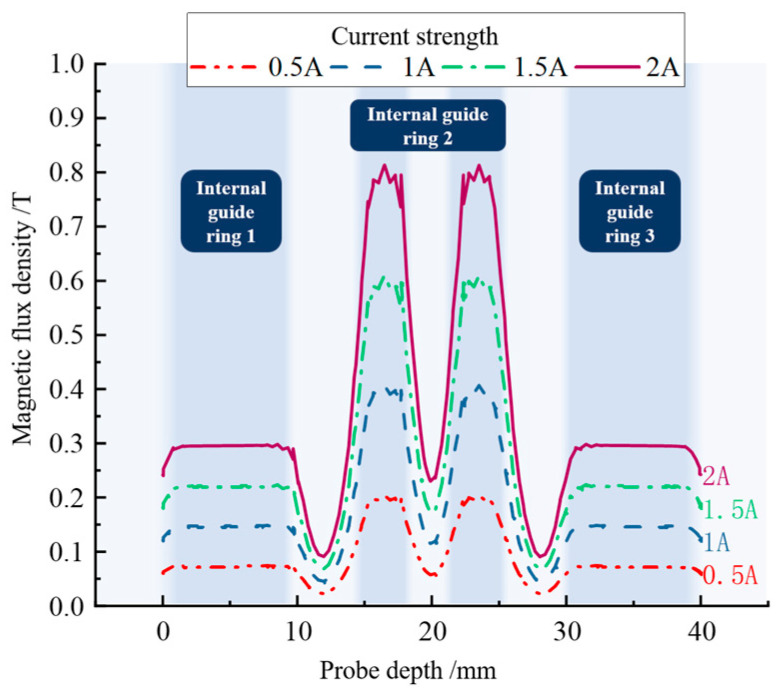
Magnetic flux density distribution simulation.

As can be seen in Figure 9, the magnetic field strength distribution varied as the probe depth varied, which is consistent with the magnetic line of force simulation results. The magnetic field intensity increased when the probe passed the position of inner guide ring 1, and decreased sharply when it passed the damping ring between guide ring 1 and guide ring 2. The magnetic field intensity increased sharply when passing through the part of the guide ring 2, which was the most concentrated location of magnetic field intensity distribution in the damping channel. When the current passed into the coil was 2 A, the induced magnetic field intensity was 0.83 T, and the magnetic rheology lipid did not reach the magnetic saturation value. The reason for this situation is that the magnetic flux was consumed in the gap of each part; according to the analysis of the magnetic field simulation diagram, there were also some magnetic inductors leaking from the MRG, the damping ring, and the outer cylinder barrel. The magnetic field in the damping channel did not reach the magnetic saturation value of the magneto-rheological grease, but the magnetic induction lines in the damping channel were increased to 1.5 times that of the conventional structure due to the bending and folding magnetic circuit design; thus, the current magnetic field strength was sufficient for the MR buffer to output the target damping force.

## 3. Buffer Performance Test

**Figure 10:**
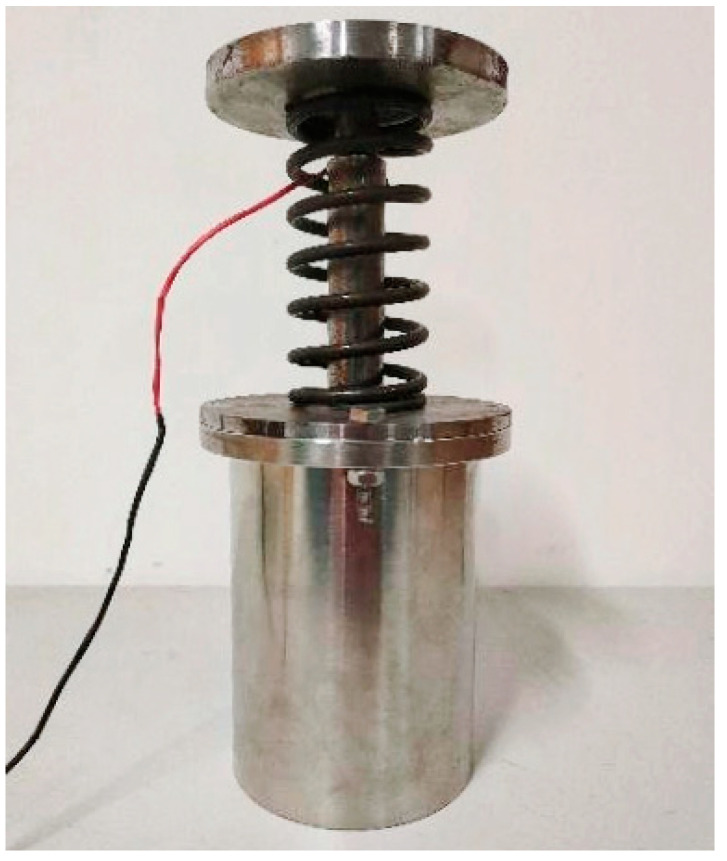
Physical diagram of the new magnetorheological buffer.

After the magnetic circuit was designed and simulated to verify the validity, the new MR buffer could be machined according to the design drawings, as shown in Figure 10.

### 3.1. Magnetic Field Strength Test

**Figure 11:**
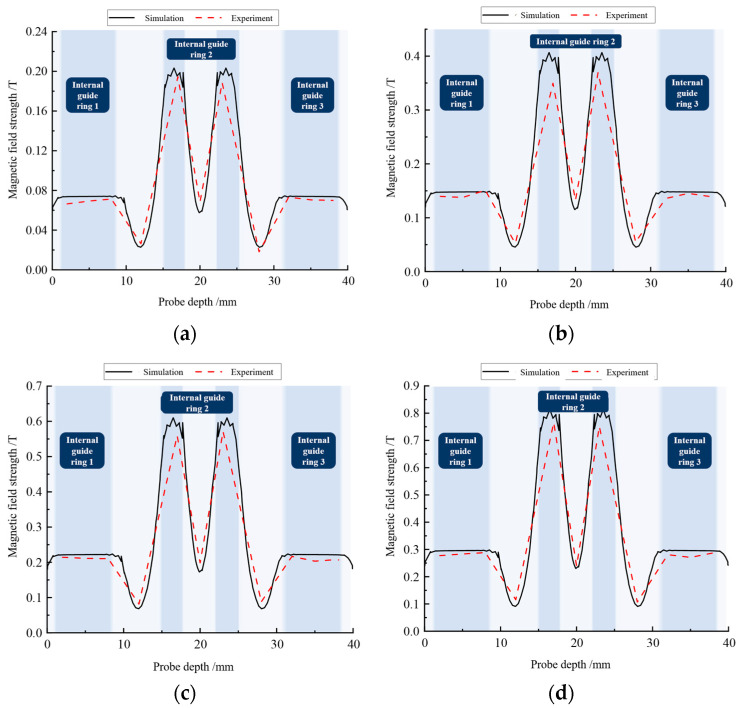
Simulation and experimental comparison of magnetic field intensity distribution in damped channel with (**a**) 0.5 A, (**b**) 1 A, (**c**) 1.5 A, and (**d**) 2 A incoming current.

A digital Tesla meter KCS-601B (Ningbo Canmag Technology Co., Ltd., Ningbo, China) was used to measure the magnetic field intensity distribution in the damped channel, and the test results were compared with the simulation results. The probe was placed in the damping channel, and the magnetic induction was recorded at 4 mm intervals from top to bottom. Figure 11 shows the comparison between the actual magnetic field strength and the simulation results for currents of 0.5 A, 1 A, 1.5 A, and 2 A.

By comparing the experimental test results with the simulation results for different excitation currents, it was found that the designed magnetic circuit meets the design requirements in terms of magnetic field intensity distribution. As the Tesla meter probe penetrated deeper and deeper into the damping channel, the magnetic field intensity changed, and the peak intensity of the magnetic field appeared around the permeable ring 2 because it was located in the dense foldback area of the magnetic induction lines of the bending foldback magnetic circuit. Further analysis shows that the magnetic field intensity in the area where the ring was located was higher than that in the area around the nonconducting ring, indicating that the main magnetic lines of force were distributed around the ring, which also verifies the effectiveness of the alternating design of the ring and the nonconducting ring.

### 3.2. Mechanical Properties Testing

**Figure 12:**
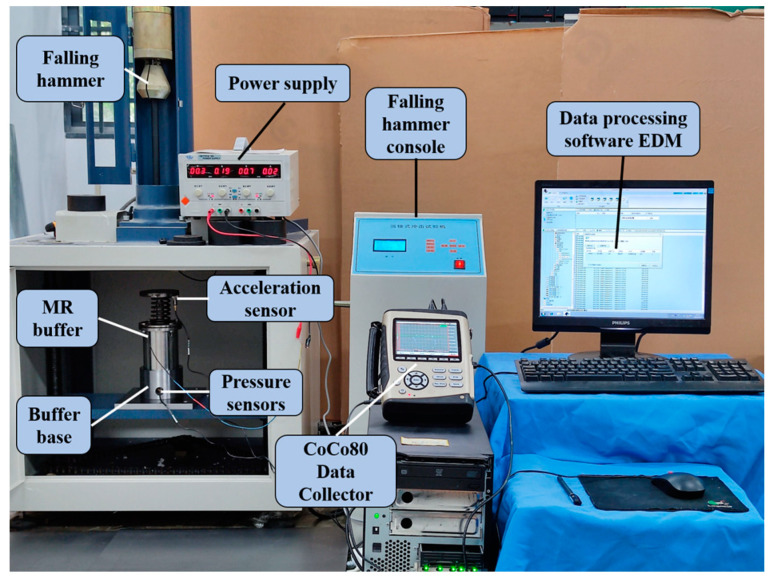
Magnetorheological buffer mechanical performance test bench.

The constructed test bench for mechanical properties is shown in Figure 12. The height of the falling hammer was set to 300 mm and the weight was 1 kg; the impact on the buffer was carried out by free-fall motion. The buffer characteristics of the buffer at each current value were studied. A DC current source YB1731A (Xiamen somai Electronic Technology Co., Ltd., Xiamen, China) was used to provide a fixed current to the magnetorheological buffer, and a CoCo80 (Crystal Instruments Co., Ltd., Santa Clara, CA, USA) digital signal collector acquired data from the acceleration sensor DH301 (Donghua Testing Technology Co., Ltd., Jiangsu, China) fixed at the top of the MR buffer and the pressure sensor 9351B (Kistler Co., Ltd., Winterthur, Switzerland) at the bottom. The data were transferred to a computer and further postprocessed using the CoCo80’s own data processing software EDM (1.3.5.6). The velocity data were obtained by integrating the acceleration; similarly, the position information was obtained by further integrating the velocity data.

#### Buffering Characteristics

**Figure 13:**
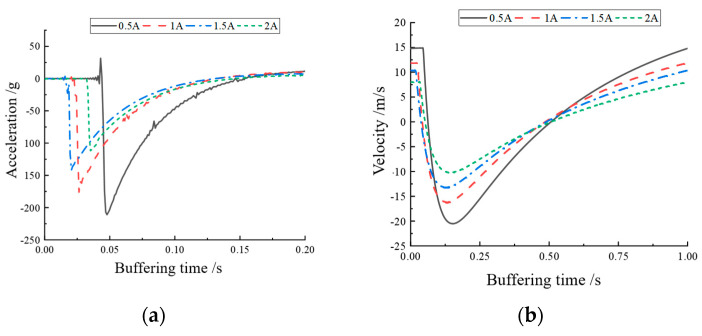

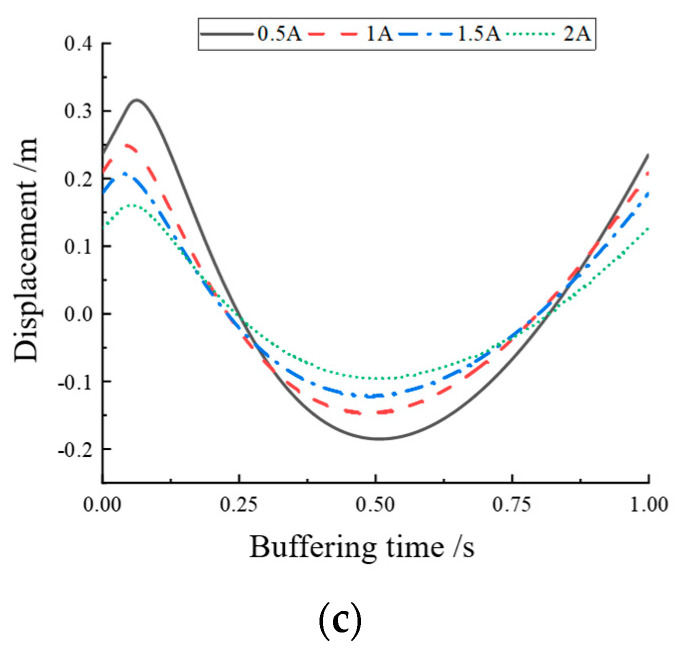
Variation of each current displacement at 300 mm height: (**a**) acceleration variation; (**b**) velocity variation; (**c**) displacement variation.

From Figure 13a, it can be seen that the response acceleration value gradually became smaller when the current increased, indicating that the vibration caused by the shock was gradually absorbed by the MR buffer as the current increased. The current curves in the figure had different starting points due to errors in the response capability of the MR buffer and the accuracy of the sensor, but this did not affect the analysis of the buffer performance. Figure 13b,c show the velocity versus time and displacement versus time for impacting the top-end cap, both obtained by integrating the acceleration variation graph. From Figure 13b,c, it can be seen that the amplitude of velocity and displacement change decreased with the increase in current, which proves that the designed MR buffer was effective in response to the shock, the designed magnetic circuit was correct, and the buffer damping force could be enhanced with the increase in current.

**Figure 14:**
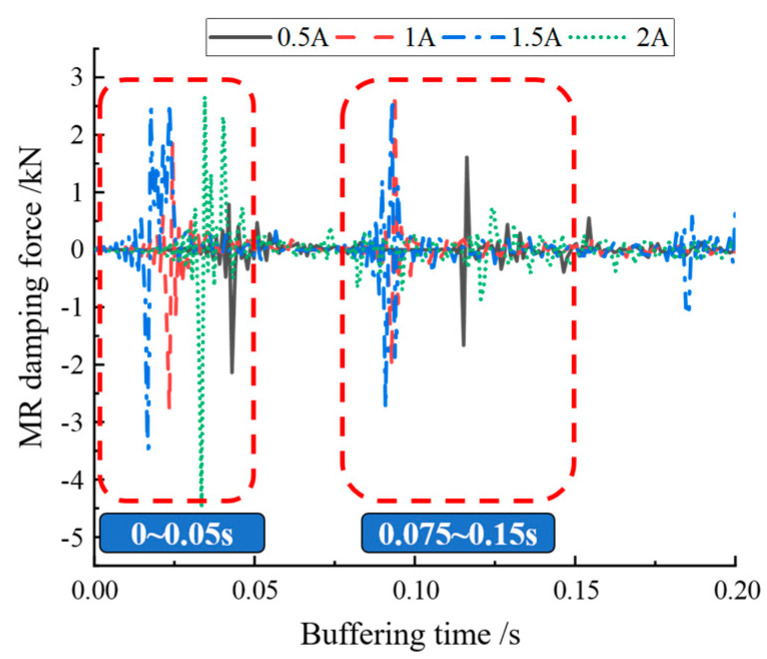
Variation of damping force for each current buffer.

**Figure 15:**
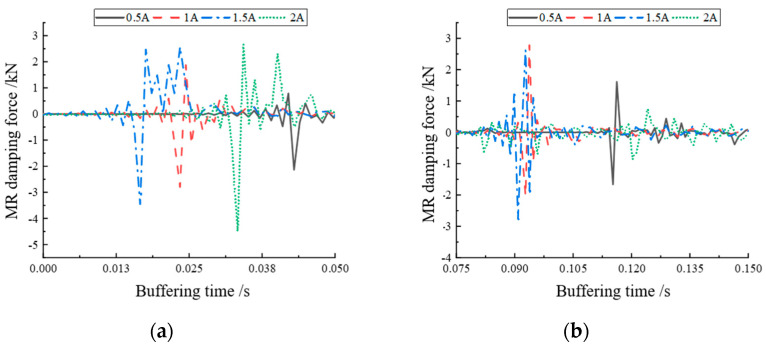
Local magnification of damping force: (**a**) comparison of damping force of each current in 0–0.05 s; (**b**) comparison of damping force of each current in 0.075–0.15 s.

Figure 14 shows the variation curves of buffer damping force at each current magnitude. The curves show that the variation of magnetorheological damping force was concentrated in the regions of 0–0.05 s and 0.075–0.15 s, which indicates that the magnetorheological buffer made a secondary jump when performing drop hammer impact buffering, putting the buffer control ability of the magnetorheological buffer to the test. The area where the buffer damping force was more concentrated was enlarged to obtain Figure 15a,b. From Figure 15a, it can be seen that the buffer damping force increased as the current increased. Similarly, the starting position of magnetorheological buffer damping force at different currents was different, but this did not affect the analysis of buffer performance. From Figure 15b, it can be seen that the magnetorheological damping force gradually increased when the current increased from 0.5 A to 1.5 A, whereas, when the current was 2 A, the magnetorheological damping force decreased sharply instead, even less than when the current was 0.5 A. This may have been caused by the fact that the damper outputted a larger damping force in the region of 0–0.05 s, which made the buffer consume more impact force. However, this may have also been due to excessive current, which caused the internal magnetorheological lipid material to become oversaturated and, thus, unable to output a sufficient amount of damping force.

### 3.3. Response Time Test

**Figure 16:**
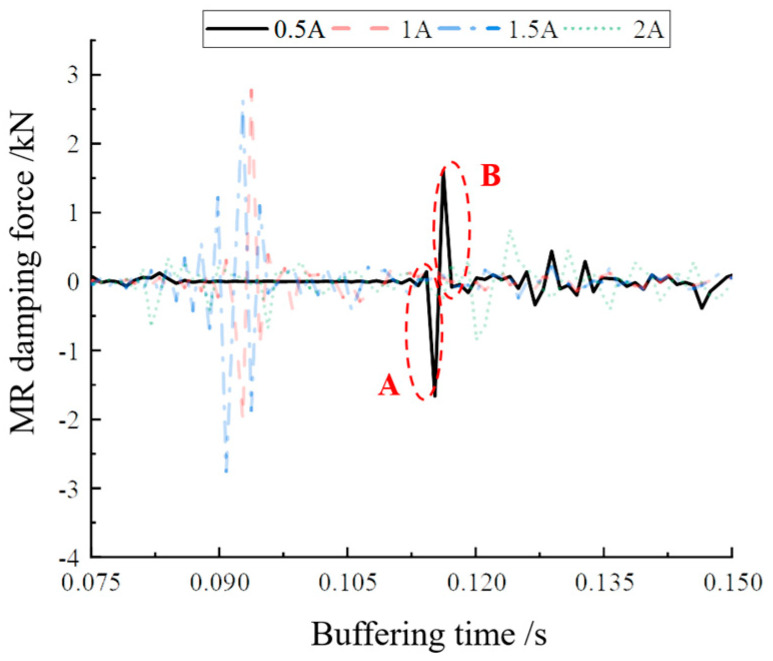
Magnetorheological damping force step part.

**Figure 17:**
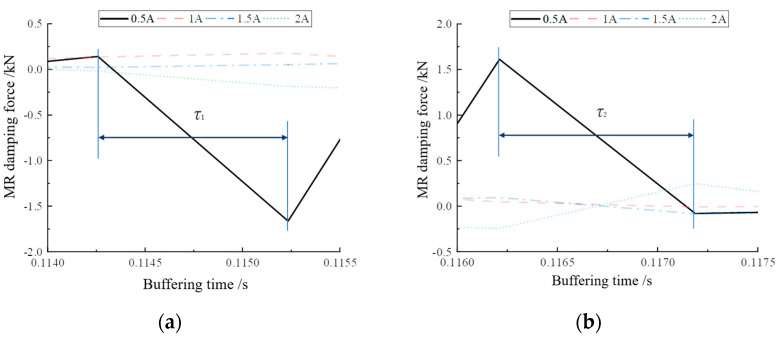
Local magnification of damping force: (**a**) magnification of damping force change in area A; (**b**) magnification of damping force change in area B.

Since there is no unified test standard for the damping force response time of MR buffers, the interval between the start of the incoming current and the output of the actual damping force is defined as the response time in this section to investigate the controlled response performance of the designed MR buffers. The mean value was calculated from the damping force change of two cycles to evaluate the real-time controlled performance of the MR buffer. The buffer damping force curve at a current value of 0.5 A was selected for the study, considering that too large a current may lead to oversaturation inside the MRG. Figure 16 shows the damping force variation curve at a current of 0.5 A at a height of 300 mm. Figure 17 shows the local enlargement of the two areas A and B.

Koo [30] proposed a method for the definition and the experimental determination of the response time of MR dampers. The response time is defined as the time required to transition from the initial state to 95% of the final state. On this basis, this paper took the jump time of the peak value of the output damping force as the response time. By averaging the damping force peak alternation times of Figure 17a,b, the damping force jump time τ_1_ from one peak to another in Figure 17a was 0.01 s, and the buffer damping force peak jump time τ_2_ in Figure 17b was 0.008 s. Therefore, it can be concluded that the damping force response time of the MR buffer was 0.009 s, i.e., 9 ms. This provides a basis for the internal damping force response time data of the magnetorheological buffer for the subsequent feasibility verification experiments of optimal buffer control.

## 4. Buffering Control Feasibility Experiments

**Figure 18:**
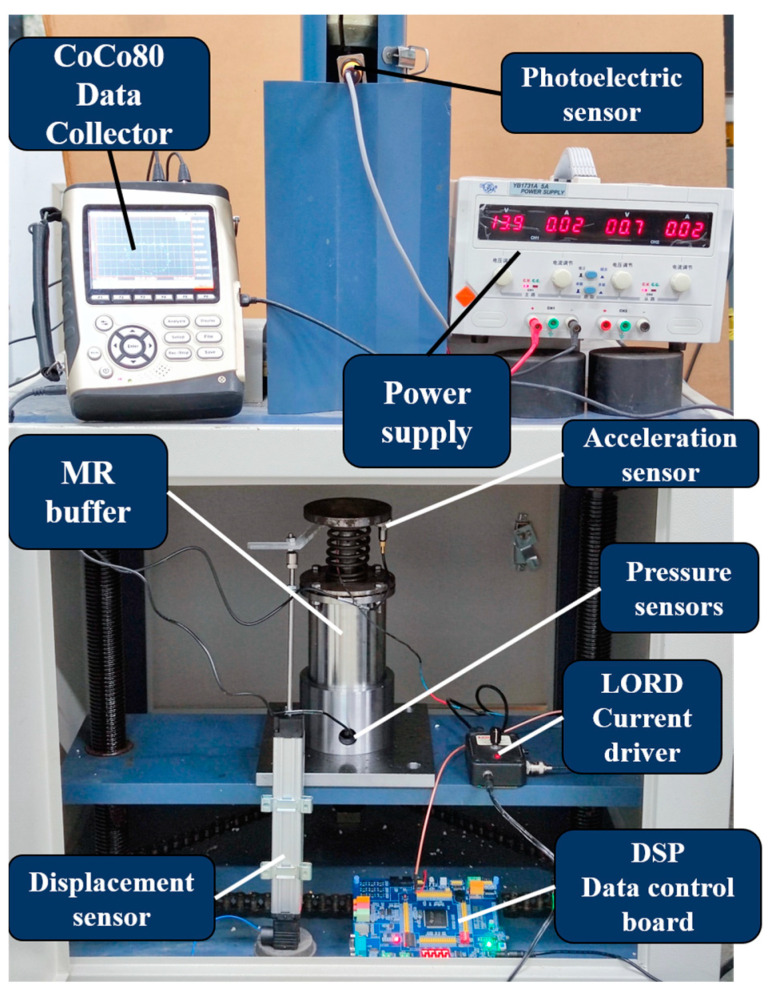
Optimal buffer control experiment bench.

The automatic variable current buffer control test bench was constructed as shown in Figure 18. The drop hammer height was set to 300 mm and the drop hammer mass was set to 1 kg, causing the MR buffer to be impacted by the free fall motion. The DC current source and CoCo80 data collector, as well as the acceleration and pressure sensor models, were the same as in Figure 12. The difference was the inclusion of the displacement sensor LWH-150 (Novetechnik Co., Ltd., Baden Werdenberg, Germany), as a more accurate amount of displacement data was required considering the need for variable current control. In addition, the need to continuously vary the current magnitude led to the inclusion of the current controller RD-3002-1 (Lord Co., Ltd., Cary, NC, USA), which can either manually turn a knob to vary the output current magnitude or control the output current value in real time with an external input voltage control signal. The maximum input/output current value was 2 A, and the input control signal could be switched up to 1 kHz.

### 4.1. Manual Variable Current Control

**Figure 19:**
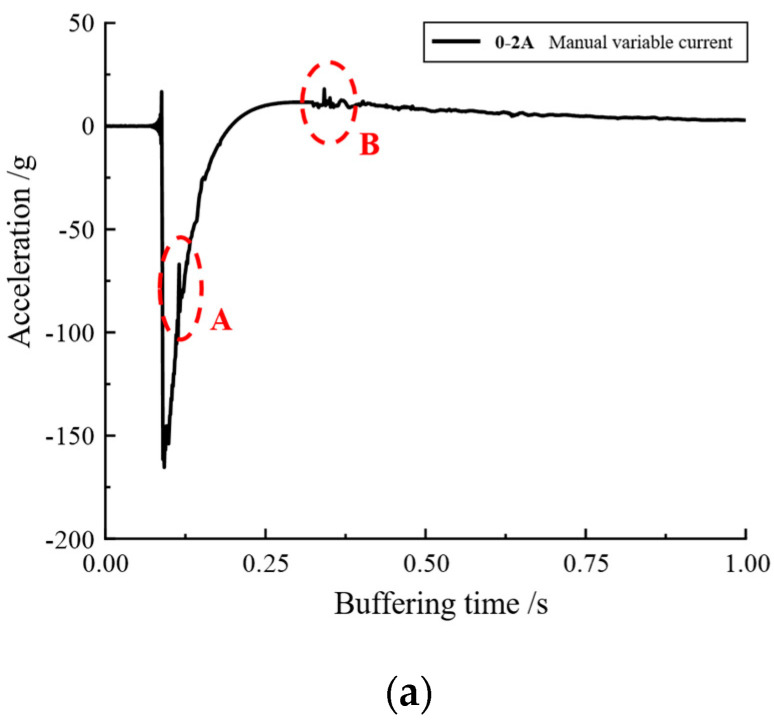

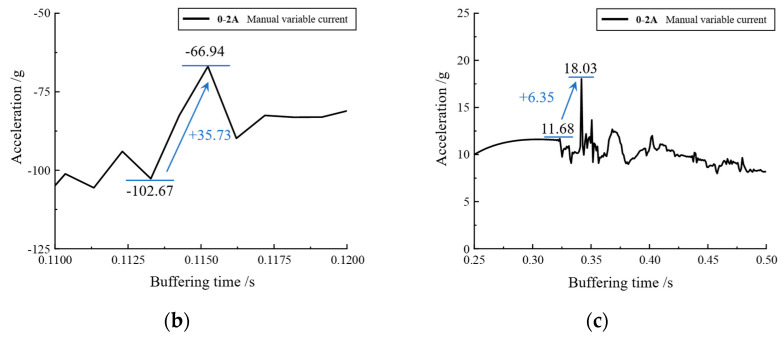
Manual adjustment of 0–2 A current magnitude: (**a**) acceleration variation curve; (**b**) acceleration variation curve partially enlarged A; (**c**) acceleration variation curve partially enlarged B.

The manual variable current buffer control experiment was first conducted to verify the feasibility of adaptive optimal buffer control. The output knob of the Lord current driver was twisted at the moment when the falling hammer was dropped, and the data with a more matching rhythm were obtained and analyzed after several experiments. Figure 19a shows the variation of magnetorheological buffer acceleration when the current was manually varied, and Figure 19b,c show the local enlargement of area A and B.

**Figure 20:**
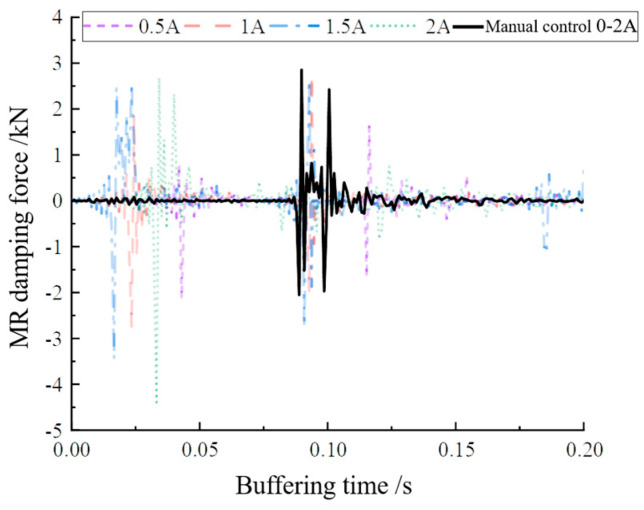
Manual variable current 0–2 A buffer damping force and each constant current buffer damping force comparison.

From Figure 19a, it can be seen that there was a brief fluctuation of acceleration values in the two regions of AB, and the local magnification yielded Figure 19b,c, which show increases of 35.73 g and 6.35 g in the fluctuation region for a short period of time, respectively. If the shock buffer experiment was conducted with a constant current, such a drastic fluctuation would not have occurred; hence, it can be assumed that the acceleration values were caused by the manual adjustment of the current magnitude. The localized sudden increase in acceleration values can be considered as a result of the manual adjustment of the current magnitude. It can be seen that the control of the buffer damping force could be achieved by the continuous variation of the current magnitude. Figure 20 shows the damping force for manually varying the current compared to a constant current.

As can be seen in Figure 20, when the manual control current was large, the buffer damping force was more concentrated compared to the constant current. Although the damping force was not as large as the peak damping force at 1 A, 1.5 A, and 2 A, the more concentrated damping force and longer buffering duration also made the buffering damping force performance of the manually controlled current better than the damping force at constant current. This is because the peak damping force was evenly distributed over the duration of the buffer, reducing the large additional momentary shock force and providing protection to the buffer.

### 4.2. Automatic Variable Current Control

**Figure 21:**
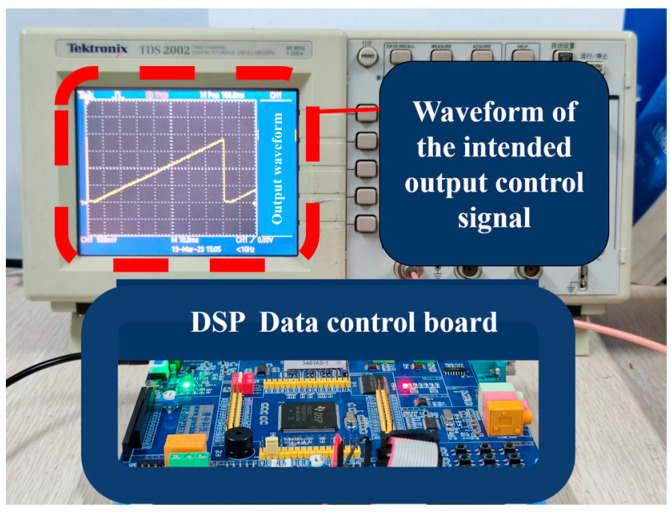
DSP output of variable current control signal.

The digital-to-analog conversion module built into the DSP processing chip outputs the control voltage to the Lord current controller, which can realize the corresponding current change curve to the magnetorheological buffer, thus achieving the purpose of automatic continuous variable current control. The variable current experiment was a feasibility test; thus, the output voltage signal could be increased linearly from small to large, as shown in the oscilloscope in Figure 21.

**Figure 22:**
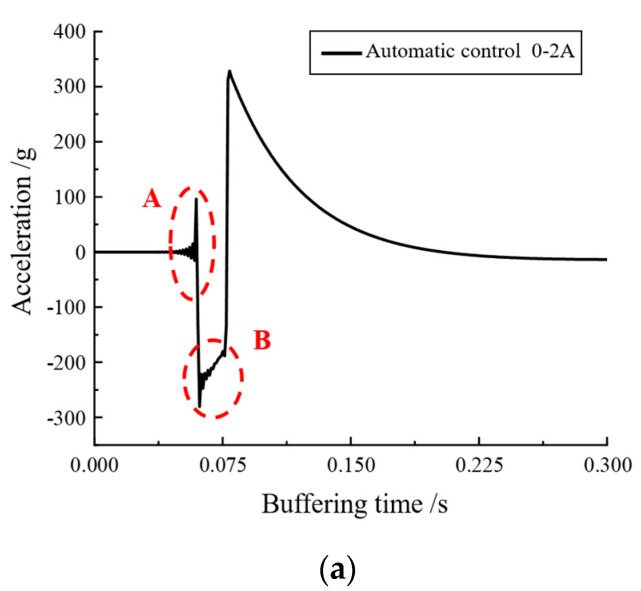

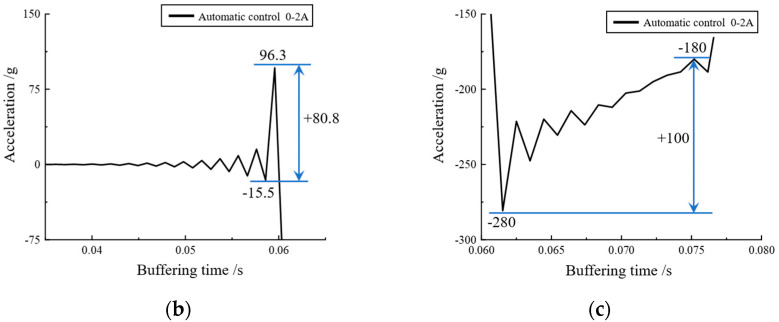
Automatic adjustment of 0–2 A current magnitude: (**a**) acceleration variation curve; (**b**) acceleration variation curve partially enlarged A; (**c**) acceleration variation curve partially enlarged B.

The designed voltage–current continuous variation signal was input into the magnetorheological buffer and a drop hammer impact experiment was conducted to record the variation curves of acceleration, velocity, displacement, and buffer damping force in order to analyze the effect of its variable current control and, thus, judge the feasibility of optimal buffer control. Figure 22 show the acceleration variation of the MR buffer and the local enlargement of the A and B regions when the current was automatically adjusted.

**Figure 23:**
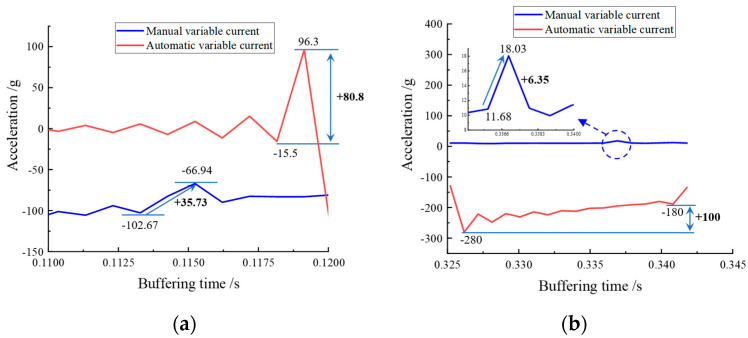
Automatic variable current control versus manual variable current control: (**a**) comparison of area A; (**b**) comparison of area B.

As can be seen from Figure 22, when the designed control signal was input, its acceleration curve underwent positive and negative fluctuations, and, when the acceleration local area A and B were enlarged, uniform fluctuations were found to increase and decrease, indicating that the automatic continuous variable current control was effective. In addition, by comparing the graph of acceleration variation during automatic current change with the manual current change control, as can be seen in Figure 23a, the acceleration change amplitude of the automatic current change control was 80.8 g, which is a significant performance improvement in terms of acceleration amplitude control compared to 35.73 g of the manual current change control. This comparison is even more evident in Figure 23b, where the amplitude of acceleration change for the automatic variable current control was 100 g, while that for the manual variable current control was only 6.35 g (which has been partially enlarged in the figure). Thus, the automatic variable current control was more effective in the control of the acceleration amplitude change.

## 5. Conclusions

This paper took the buffer system combined with magnetorheological buffer and spring as the research object, and completed the structural design, dynamic characteristic analysis, and performance test of magnetorheological buffer with a bending foldback magnetic circuit, as well as completed the assembly, simulation analysis, and buffer control feasibility experiment of the magnetorheological buffer system. The main research results and conclusions are as follows:

(1) By improving the magnetic circuit distribution, the magnetic lines of force were interspersed in the damping channel several times to improve the utilization rate of magnetic induction lines. The magnetic field analysis results of Comsol (5.6) simulation software showed that the magnetic circuit of the proposed magnetorheological buffer was bent and folded back to increase the effective area of the damping channel, and the actual available magnetic induction lines were 1.5 times of the traditional buffer.

(2) An experimental study on the energy absorption characteristics and optimal controllability of the shock magnetorheological buffer system was conducted. The designed magnetorheological buffer was machined and assembled, and its magnetic field strength was tested and compared with the simulation results to test the mechanical properties and current response time. An experimental bench was set up to analyze the cushioning effect under a 300 mm drop height. The experimental results showed that the designed magnetorheological cushioning system could reduce the displacement and acceleration response of the cushion piston caused by the impact of the falling hammer more effectively than the conventional damping control at a height of 300 mm and a current of 0–2 A. This provides a reference for the feasibility of the magnetorheological optimal cushioning control technique under impact load.

## Figures and Tables

**Table 1:** Main parameters of magneto-rheological grease.

Main Parameters	Value
Density	6.5 g/cm^3^
Viscosity (γ = 10 s^−1^, 10 °C)	8 Pa·s
Flux saturation density	1.31 T
Operating temperature	−40 to 130 °C

**Table 2:** Dimensional parameters of magnetorheological buffer components.

Parameters	Value (mm)
Top end cap diameter	100
Inner diameter of cylinder barrel	70
Cylinder wall thickness	10
Piston diameter	45
Piston coil groove length	25
Piston coil groove width	10
Inner guide ring diameter	58
Inner resistance ring diameter	58
Outer ring diameter	70
Outer resistance ring diameter	70
Damping clearance	2
Piston rod diameter	20
Piston rod length	130

## Data Availability

The data that support the findings of this study are available on request from the corresponding author (yangqing@zjxu.edu.cn, hhs999@mail.zjxu.edu.cn).
